# The yips: an investigation of the causes and treatments in the context of golf

**DOI:** 10.3389/fspor.2025.1563370

**Published:** 2025-04-17

**Authors:** Kian-Taj Claire Afrassiabian, Bisundev Mahato, Ulrick Vieux, Nikhil Palekar

**Affiliations:** ^1^ Department of Psychiatry, Castle Point VA Medical Center, Wappingers Falls, NY, United States; ^2^ Department of Psychiatry, Garnet Health Medical Center-Catskills, Harris, NY, United States; ^3^ Department of Psychiatry, Nilux Clinic, Hollywood, CA, United States; ^4^ Department of Psychiatry, Nilux Clinic, Manhattan, NY, United States; ^5^ Department of Psychiatry, Hackensack Meridian Health-Hackensack University Medical Center, Hackensack, NJ, United States; ^6^ Department of Psychiatry, Stony Brook University Hospital, Stony Brook, NY, United States

**Keywords:** yips, golf, sports, movement, performance

## Abstract

**Aim:**

When a skill is lost, this can impact a person's career and psychological wellbeing. As this is the case with the yips, preventing, treating, and curing them is important. This study was prompted by the limited information available on the yips, specifically regarding etiologies. The intent of this study was to utilize surveys with both a medical review of systems and psychological questioning to identify commonalities in afflicted players. This could then provide considerations of the causes and guide future research.

**Methods:**

The study recruited experienced golfers for in-person surveys and stroke demonstrations from January 26, 2019 to March 28, 2020. The analyses included statistical methods and discussion. These methods included Yates’ chi-squared test and Fisher's exact test targeted toward smaller sample sizes.

**Results:**

Finding participants was difficult and resulted in a small sample size (*n* = 14). The study had 4 participants with the yips and 10 controls. Statistical methods for small sample sizes found few associations. However, Fisher's exact test showed negative associations between the yips and sore throats (*p* = 0.0050), fever (*p* = 0.0150), and physical trauma (*p* = 0.0150). Yates' chi-squared test also found significance for fever (*p* = 0.0328), sore throat (*p* = 0.0105), and physical trauma (*p* = 0.0328). Qualitative data were also highlighted in the study, including tabulating the treatments tried and the outcomes of the participants.

**Conclusion:**

The yips cause problems, especially for those who have invested significant time into perfecting a skill. Continued research is warranted to elucidate the causes and effective treatments.

## Introduction

The yips are unintended movements that impact accuracy. Their onset is sudden, with no apparent explanation or foreshadowing. In golf, they appear as involuntary movements and/or freezing during a stroke (1–6). These movements have been described as jerking, twisting, and/or a tremor, while freezing is a hesitation prior to executing a swing (1–6).

The yips have been categorized in a variety of ways. They have been described as “physical symptoms (dystonia: type I), psychosocial symptoms (choking: type II), or a combination of both (non-categorized)” (6). One study categorized the yips as dystonic and non-dystonic where dystonic yips had a “jerk, twist, or posturing” (7), and non-dystonic forms appeared as “pushing or steering” the ball while putting (7). Similarly, other research has highlighted a cramping/dystonia perspective (1–3). The prevalence of perceived yips in studies has varied based on sample size. In a 69-question survey provided to 1,050 “professional and amateur golfers,” 42% responded, of which 28% self-reported having the yips (1, 2). In a separate survey sent to 2,630 tournament players, 1,031 (39%) responded and 52% felt they had the yips (5). Furthermore, they have been associated with older age (1, 2). Outside of sports, they afflict musicians, surgeons, and dentists (1, 2, 4, 6).

Regarding the psychological aspects of the yips, the topic of anxiety is frequently addressed. Researchers have found that the yips may be exacerbated by performance anxiety, a fear associated with a specific task, and “choking” (4, 6). Choking has been a focal point of some research and is described as “an extreme manifestation of performance anxiety” and “a suboptimal performance that results from self-focused or distracted attention” (6). One study concluded anxiety is not a sole cause, but “a necessary factor for the manifestation of the yips in a number of golfers, and exacerbates the problem in others” (4).

There have also been neurological studies conducted on the yips, many of which sought to better identify them and liken them to movement disorders. Thus, the topic of dystonia was added to the discussion. In one approach, the yips were delineated into neurological (dystonia) and psychological (choking) categories (6). Dystonia, “a neurological disorder characterized by involuntary movements resulting in spasms, twisting and posturing of a body part,” has described the yips and some suggest they are a “task-specific dystonia” (6). It was postulated for dystonic types that “performance anxiety contributes to and exacerbates the ‘yips’ symptoms” (6). For “chokers,” performance anxiety likely combines “with self-focused attention and over-analysis to cause the ‘yips’” (6). This study implied the unlikeliness of dystonic or choking symptoms occurring in the “absence of performance anxiety” (6).

The yips have destroyed the careers of many professional athletes, causing them to either circumvent their damaged technique using a lesser form or abandon their sport. While there are no FDA-approved medications for the yips, the suspected contributing factors such as anxiety have known protocols. Propranolol and diazepam are two of these that have been prescribed off-label (8). If cramping/dystonia is present, botulinum toxin injections have been prescribed (1–3). Some suggest taking a break, and instructors teach new techniques to change muscle memory.

The task of this pilot study is to further explore potential medical and psychological etiologies of the yips and their treatment in the context of golf.

## Methods

### Recruitment

Participants were solicited for a telephone prescreening via postings at local golf facilities, classified ads in a local newspaper, emails, and word of mouth in the Hudson Valley region of New York and southeastern Pennsylvania. Emails were also specifically sent to Professional Golfers’ Association of America (PGA) players in an effort to recruit competitive tournament players. If the criteria were met, an in-person survey and stroke demonstration were arranged locally. A witnessed signing of an informed consent form, as required by the Internal Review Board that approved this study, was completed upon meeting.

The inclusion criteria for this study included significant golf experience (i.e., tournament experience and/or 18-hole competition >3 years) and age (18–80 years). This experience was validated in discussions with players and reviewing their competitive histories online. This age range was sought to exclude potential inexperience in younger players, particularly in regard to learning a stroke and recognizing developmental changes in youth (i.e., growth spurts). In addition, confounding medical comorbidities in elderly adults were considered when choosing the age limit.

The exclusion criterion was a current movement disorder diagnosis. We separated participants into two groups: yips and no yips (controls). The inclusion criterion for a yips group participant (YGP) was a “yes” response to all the following screening questions:

1. Have you developed a significant difficulty with a golf swing (putting, driving, chipping, etc.) that was once an asset in your game?
2. Have you noted any form of tremor, involuntary movement, jerking, twisting, and/or freezing (pausing) develop in your stroke (1–7)?
3. Have you found difficulty in recovering that golf swing to its original effectiveness?

### Survey

The 156-question survey consisted of medical review of systems (ROS) questions (digestive, immune, respiratory, etc.). The goal was to employ the basics of a medical investigation to discover connections between the yips and a system. Additional categories included psychiatric/psychological, infection/illness, trauma, coaching, treatment, medications, yip description, family history, general health, yip history, and personal opinion. The questions were organized into subcategories based on their medical system. These surveys were conducted in-person and based on the participants’ accounts of their medical and psychological histories. The participants were required to answer all questions in the musculoskeletal, cardiac, neurologic, respiratory, and psychological subcategories; however, if they denied diagnoses in other medical categories, they were not required to answer questions under that system. This helped avoid fatigue, and differences were accounted for in statistical measures.

### Observations

Each participant demonstrated a stroke (chipping, putting, or driving) to confirm the presence of the yips or not. Players were asked to demonstrate their golf strokes three times (in person) to ensure they were properly warmed up and appropriately replicated. Observations for the yips movements included jerking, twisting, and/or posturing patterns in comparison to standard stroke accordance.

### Data collection

Survey responses were organized and analyzed using Microsoft Excel. The website langsrud.com (9) was used to calculate Fischer's exact test values.

### Data analyses

Yates' chi-squared test and Fisher's exact test were the statistical methods used. These were chosen as they are intended to work with smaller sample sizes to identify statistically significant data from them.

In Fischer's exact test, if a one-tailed left test (negative relationship) accompanied a statistically significant “two-tailed test,” this suggested a negative relationship and, conversely, should a one-tailed right test accompany a two-tailed test, this suggests a positive relationship. Yates’ chi-squared test was another statistical assessment utilized to confirm the relationships.

Survey questions on popularized or potentially relevant topics of yips etiologies were chosen for presentation in charts/tables and the discussion.

## Results

Surveys were administered from January 26, 2019 to March 28, 2020 and 14 participants volunteered. Four were YGPs and 10 were controls. Difficulties finding participants resulted in a small sample size. There were 13 men and 1 woman (control) in the study (Table 1). There were limited responses to outreach attempts, which included flyers in newspapers, postings in private golf clubs, and emails to PGA players.

**Table 1:** Participant demographics.

Code	Sex	Age	Yips	Controls
1	M	55	Yes	
2	M	29		X
3	M	56		X
4	M	55		X
5	F	62		X
6	M	63		X
7	M	58		X
8	M	34		X
9	M	26		X
10	M	26		X
11	M	57		X
12	M	74	Yes	
13	M	68	Yes	
14	M	54	Yes	
		51.2	62.8	46.6
		Total average	Average with the yips	Average no yips

The above data identify the participants’ age, gender, and whether the yips were present.

X, control participants that did not have yips.

The majority of the ROS and psychiatric/psychological questions proved statistically insignificant for both Yates’ chi-squared test and Fisher's exact test. However, negative associations existed between the yips and infections involving sore throats (*p* = 0.0050) and fevers (*p* = 0.0150) (Table 2). If a one-tailed association (positive or negative) accompanied a two-tailed association, this was significant. The trauma-related question, “Have you endured any physical trauma prior to the development of your yips (i.e., car accident, falling on your arm)?”, was also negatively associated (*p* = 0.0150) (Table 2). Yates' chi-squared test also helped confirm these relationships involving sore throats (*p* = 0.0105), fever (*p* = 0.0328), and any physical trauma (*p* = 0.0328).

**Table 2:** Yates' chi-squared test and Fisher's exact test results for the infection, trauma, and coaching questions.

Question topic	Fisher's exact test *p*-values			
	Yates's chi-squared test	one-tailed left	one-tailed right	Two-tailed test
	*p*-value 1 degree of freedom	Negative relationship	Positive relationship	
Fever	**0**.0328*	**0**.0150*	1.0000	**0**.0150*
Sore throat	**0**.0105*	**0.0050** *	1.0000	**0**.0050*
Food poisoning	0.1047	0.0490*	1.0000	0.0699
Diarrhea	0.1466	0.0699	1.0000	0.0849
Abrasion	0.1466	0.0699	1.0000	0.0849
Any infection	0.3414	0.1762	1.0000	0.2280
Diagnosis infection/illness	0.3414	0.1762	1.0000	0.2280
Travel overseas	0.3999	0.2098	1.0000	0.2507
Any trauma	**0**.0328*	**0**.0150*	1.0000	**0**.0150*
Change stroke/grip	0.5541	0.2797	0.9650	0.5594
Change coach	0.7119	0.6667	1.0000	1.0000

The data above were created from questions in the infection, trauma, and coaching sections of the survey. Statistical analysis conducted via Yates' chi-squared test showed significance for fever with a *p*-value (1 degree of freedom) of 0.0328 (*n* = 14), sore throat with 0.0105 (*n* = 14), and any trauma with 0.0328 (*n* = 14). Statistical analysis conducted via Fisher's exact test (one-tailed left) showed significant negative relationships for fever with a *p*-value of 0.0150 (*n* = 14), sore throat with 0.0050 (*n* = 14), and any trauma with 0.0150 (*n* = 14). The Fisher's exact test (two-tailed) results were significant, confirming associations for fever with a *p*-value of 0.0150 (*n* = 14), sore throat with 0.0050 (*n* = 14), and any trauma with 0.0150 (*n* = 14). Values listed in bold are statistically significant.

* *p* < 0.05.

Regarding some specific findings, 3/4 (75%) of the YGPs felt the yips were not preventable. The YGPs thought they were psychological in nature and age-related. Furthermore, 3/4 (75%) of the YGPs sought treatment including but not limited to changing grips, sports psychology videos, playing with their non-dominant hand, and using long putters and/or wedges for sensory distraction purposes. Only 1/4 (25%) thought medication could be related to golfing issues. Half (2/4) of the YGPs and 3/10 of the controls had taken anti-anxiety medication, including diazepam, alprazolam, and beta blockers. Botulinum toxin injections were not mentioned.

Half (2/4) of the YGPs self-medicated using alcohol and found no benefit. In addition, 3/4 (75%) of the YGPs and 5/10 (50%) of the controls had sleep issues. Although prior musculoskeletal trauma was not indicated by the YGPs, 2/4 (50%) had surgery after acquiring the yips. Interestingly, one surgery involved wrist carpel tunnel syndrome, and the other trigger finger release surgery. Concerning coaching, 1/4 (25%) of the YGPs attempted to change their grip/stroke technique as advised prior to developing their yips. All the YGPs indicated decreased performance, but only 2/4 (50%) said someone else noticed their yips, inclusive of golf peers and coaches. A list of strategies attempted by the YGPs to overcome the yips and their outcomes are presented in Table 3.

**Table 3:** Verbal survey responses concerning the techniques and treatments attempted and their outcomes.

Unreported outcome	Helped	Did not find helpful
Technique	Technique	Technique
Using non-dominant hand	One-handed putting	Putting left-handed
Blocking the hole	“Plug the hole”	Different physical grips
Changed grip	Cross-handed	One-handed chip
Different grips	Cross-handed putting	Non-cross-handed chip
Medical	Cross-handed chipping	Different forms of putting
Eye doctor	Putting with eyes closed	Claw grips
Hypnosis	Cross-handed putting with a long putter	Medical
Equipment	Putting with non-dominant hand	Eye doctor
Bigger head	Medical	Sports psychologist
Bigger grip	Sports psychologist	Equipment
Training	Hypnosis professional	Fat grips
Golf pro	Self-hypnosis	Medication
Playing less	Equipment	Diazepam
Playing more	Long putter	Alprazolam
Medication/self-medicating	Long wedge	Medications
Beta-blocker propranolol	Authors	Authors
Alcoholic beverages	Fluid motion factor—Steven Yellin	Specific Golf Pro's book
Environment	Quiet mind golf—Steven Yellin	Mental
Quit tournament golf, only plays socially	Neuro-linguistic programming—Richard Bandler	A mindset strategy
Mental	Tony Robbins	
Try to return to original muscle memory by “fighting through it”	Marius Filmalter instructional videos	
	Diet	
	No caffeine	
	Balanced diet	
	Bananas	
	Low sugar	
	Education	
	Studying the brain	
	Exercise	
	Yoga	
	Environment	
	Certain settings	
	Mental	
	Changing focus—deemphasizing the importance of the outcome, i.e., money	

The table lists what was attempted to treat the yips by participants and their outcomes. Unreported outcomes are treatments where outcomes were not disclosed.

## Discussion

The lack of participants (1 woman, 13 men) limited the study, especially regarding statistical analyses. Nevertheless, a platform was developed using Microsoft Excel, and statistics were calculated, charted, and tabulated. Therefore, the major outcome of this study was building an initial prototype for future research.

There were limited responses to recruitment attempts. Some players cited concerns with discussing the yips, believing they are psychological in nature and fearing they could acquire them through discussions.

The in-person aspect of this study was important not only for data accuracy and insight but also to confirm the yips. It is not uncommon to blame a bad stroke on the yips.

The average age of yips onset was 34.8 (SD 14.62), and the participants had endured them for 40, 49, 18, and 4 years, respectively. All were seasoned tournament players at the onset. Despite 3/4 (75%) of the YGPs enduring more losses and 2/4 (50%) avoiding their bad strokes, they still enjoy golf.

Standardizing testing remains critical in observation assessment and an ongoing goal of future studies. Recent research has highlighted measuring “external load” across different training modalities (10). This concept should be considered in future analyses to better understand how load difference and exertion can contribute to flawed strokes and yips development (10).

Interestingly, two controls reported previously overcoming the yips. One reported using a 3D simulator to observe and make corrections and the other relaxed using deep breathing techniques. However, their past yips could not be verified. One research study reported a player overcame their yips in a year by changing their grip (4). These accounts suggest the yips are curable.

In conclusion, physicians need to be aware of the yips and be empathetic. These patients need compassion and understanding. There may be a tendency to make light of a bad golf swing, but this should be avoided. Prior to assuming the yips exist, this must be confirmed by ruling out movement disorders including essential tremor or Parkinson's disease and patients must be educated on the prognosis possibly being a lengthy process.

Depression and anxiety also need to be addressed as they could be the most damaging. Furthermore, the patient's support system should be assessed and kept involved. Whenever a patient working in these fields reports losing fine motor skills, the yips should be considered as a possible differential diagnosis (11).

## Data Availability

The original contributions presented in the study are included in the article, further inquiries can be directed to the corresponding author.
